# Mitochondrial and Ubiquitin Proteasome System Dysfunction in Ageing and Disease: Two Sides of the Same Coin?

**DOI:** 10.3390/ijms160819458

**Published:** 2015-08-17

**Authors:** Jaime M. Ross, Lars Olson, Giuseppe Coppotelli

**Affiliations:** Department of Neuroscience, Karolinska Institutet, Retzius väg 8, Stockholm 171 77, Sweden; E-Mail: lars.olson@ki.se

**Keywords:** ageing, mitochondria, ubiquitin, proteasome, ROS

## Abstract

Mitochondrial dysfunction and impairment of the ubiquitin proteasome system have been described as two hallmarks of the ageing process. Additionally, both systems have been implicated in the etiopathogenesis of many age-related diseases, particularly neurodegenerative disorders, such as Alzheimer’s and Parkinson’s disease. Interestingly, these two systems are closely interconnected, with the ubiquitin proteasome system maintaining mitochondrial homeostasis by regulating organelle dynamics, the proteome, and mitophagy, and mitochondrial dysfunction impairing cellular protein homeostasis by oxidative damage. Here, we review the current literature and argue that the interplay of the two systems should be considered in order to better understand the cellular dysfunction observed in ageing and age-related diseases. Such an approach may provide valuable insights into molecular mechanisms underlying the ageing process, and further discovery of treatments to counteract ageing and its associated diseases. Furthermore, we provide a hypothetical model for the heterogeneity described among individuals during ageing.

## 1. Introduction

An increase in the average age of the world population has heightened the interest in ageing research in order to find treatments to improve health in old age. However, despite vast scientific efforts, the mechanisms that regulate ageing remain poorly understood. Outstanding questions include when the process starts and how it proceeds, why different species age at different rates, and why even individuals within the same species age differently. Ageing is a complex process, including genetic and environmental factors, both with stochastic components, all concurring and integrating in a manner difficult to predict. In a recent review, López-Otín and colleagues underlined nine hallmarks of ageing: genomic instability, telomere attrition, epigenetic alterations, loss of proteostasis, deregulated nutrient-sensing, mitochondrial dysfunction, cellular senescence, stem cell exhaustion, and altered intercellular communication [1]. Notably, such putative hallmarks are not isolated cellular processes but are highly interconnected. In order to properly understand the ageing process and to identify therapies to combat ageing, the role and interconnectedness of the putative hallmarks must be further dissected.

Impairment of the ubiquitin proteasome system (UPS) and mitochondrial dysfunction are two hallmarks of ageing and both have been implicated in a plethora of ageing-associated diseases, such as Alzheimer’s and Parkinson’s disease and certain cancers [1,2,3,4,5,6]. UPS is part of the “proteostasis network” (PN), and together with the autophagy lysosome system (ALS) and the molecular chaperone network contribute to maintaining cellular protein homeostasis by removing unwanted or damaged proteins that could aggregate and become toxic for the cell [7,8,9,10]. Mitochondria are the main source of energy production, generating ATP through oxidative phosphorylation (OXPHOS), and are also involved in many other important cellular processes, such as calcium buffering, apoptosis, steroid synthesis, and reactive oxygen species (ROS) production [11,12,13]. Although mitochondria are equipped with several mechanisms to quench free radicals, they are still subject to oxidative damage and thus rely on the UPS along with other quality control mechanisms to remove damaged mitochondrial proteins. Hence, an efficient UPS is crucial to preserve healthy mitochondria, and *vice versa*, healthy mitochondria are needed to maintain an efficient UPS system, since excessive ROS production could not only overflow the proteasome by increasing the amount of damaged proteins to be removed, but could also oxidize and damage the proteasomal subunits themselves and thereby decrease their catalytic activities. Once either mitochondrial dysfunction or proteasomal impairment develops, a vicious cycle may start, leading to progressive failure of both systems. Here, we summarize current knowledge of the interplay between the two systems, underlining how they affect each other in health, ageing, and disease, as well as how therapies targeting one deficiency might also benefit the other.

## 2. The Ubiquitin Proteasome System

The discovery of the ubiquitin-mediated protein degradation system earned Aaron Ciechanover, Avram Hershko, and Irwin Rose the 2004 Nobel Prize in Chemistry. Before uncovering the UPS, protein degradation was thought to occur mainly in the lysosome, an organelle filled with hydrolytic enzymes with an optimal proteolytic activity at a low pH [14]. Proteasome-mediated protein degradation differs from lysosomal-mediated proteolysis by operating at a neutral pH, mainly degrading short-lived proteins, taking place in a protein complex, and by not involving intracellular compartmentalization. The conjugation of a polyubiquitin chain is an essential step to target unwanted or damaged proteins for proteasomal degradation [9]. Proteasome activity generates small peptides that are further digested into amino acids by the abundant cytosolic endopeptidases and aminopeptidases, while lysosomal degradation directly produces single amino acids [15]. The UPS is a highly selective system and operates in both nuclear and cytoplasmic compartments. Conversely, lysosomes are present only in the cytoplasm and are able to remove a wide range of substrates, ranging from a single protein delivered to it via chaperone-mediated autophagy (CMA) to large aggregates and whole organelles (e.g., mitochondria) engulfed via macroautophagy [16,17].

Ubiquitin [Ub] is a 76 amino acid ≈8 kDa protein that is highly conserved among Eukaryota [18,19]. Protein ubiquitination is an ATP dependent process that occurs through a three-step sequential enzymatic cascade performed by the ubiquitin-activating enzyme (E1), ubiquitin-conjugating enzyme (E2), and ubiquitin ligase (E3). The result generates an isopeptidyl bond between ubiquitin at glycine 76 and either the ε-amino group of an internal lysine residue on the protein substrate or its amino terminus. Subsequently, multiple rounds of ubiquitination extend the ubiquitin chain by adding more ubiquitins on one of the seven internal lysine residues (Lys 6, 11, 27, 29, 33, 48 and 63) of the previously added ubiquitin, which generates polyubiquitin chains with different linkages (e.g., K48, K63, *etc.*) [20]. The length and type of the ubiquitin chain determine the fate of the ubiquitinated protein; the K48-linked polyubiquitin chain is the main signal that targets substrates for 26S proteasome degradation, while other types of linkages have been shown to play a role in receptor signaling, endocytosis, transcription, DNA repair, and autophagy [21]. The E3 ligase enzyme confers specificity to the ubiquitination system by recognizing the target’s substrate; indeed, while there is one type of ubiquitin-activating E1 enzyme (ubiquitin-like modifier-activating enzyme 1, UBA1) present in all cells and a second E1 type (Ubiquitin-activating Enzyme 1-like 2, UBE1L2) with seemingly more tissue specificity [22], there are about 30 E2 enzymes and more than 600 members of the E3 family. E3 ligase enzymes can be grouped into two classes: those that are homologous to the E6-AP carboxyl terminus (HECT) and the really interesting new gene [RING] ligases. The two classes differ not only in their structure but also in the way they catalyze the last step of ubiquitination. The HECT ligases accept the activated ubiquitin from an E2 enzyme on a cysteine residue in the active domain and then transfer it to the substrate, whereas the RING ligases act as scaffold proteins by bringing together an E2 conjugating enzyme and the substrate [23].

Ubiquitination is a reversible post-translational modification, and a family of proteases, the deubiquitinating enzymes (DUBs), can remove ubiquitin from substrates, thereby regulating the ubiquitination process and recycling ubiquitin. DUBs are highly specific and have been grouped into five subfamilies: Ub carboxyl-terminal hydrolases (UCH), Ub-specific proteases (Usp), ovarian tumor like proeases, JAB1/MPN/Mov34 (JAMM/MPN) metalloproteases, and the Machado–Jakob disease proteases. Removal of ubiquitin adducts from the substrate is a critical step for proteasomal degradation [24,25].

The 26S proteasome is a multi-subunit holoenzyme of ≈2.5 MDa, with two distinct subdomains, a 20S core particle (CP) and, in the classical conformation, either one or two 19S (PA 700) regulatory particles (RP) on either side of the CP. The CP is a barrel-shaped complex made by two α- and two β-rings, each containing seven subunits (α_1–7_ and β_1–7_), and arranged with two β-rings in the middle and two α-rings on either side. The proteolytic activity is carried out by three β subunits (β1, β2, β5), each with different amino acid specificity, caspase-, trypsin-, and chymotrypsin-like activity, respectively [26]. The α subunits seem to have a regulatory function, allowing only unfolded substrates access to the inner chamber, where the proteolytic activities are located, thus avoiding non-specific degradation of cellular proteins. Ubiquitinated substrates are docked and unfolded by the 19S RP, which can be functionally subdivided into a base and a lid [27]. The base consists of six AAA-ATPase rings (Rpt1-6) and three non-ATPase subunits (Rpn1, Rpn2, Rpn13), while another subunit, Rpn10, seems to associate with the base and the lid after their assembly. The AAA-ATPases use energy to unfold the substrate and translocate it through the central pore of the 20S chamber, while two of the non-ATPase subunits (Rpn10, Rpn13) serve as ubiquitin receptors [28,29,30,31,32]. The lid has more than nine proteins, including the deubiquinating enzyme Rpn11, which is essential for efficient substrate degradation [33]. Other regulatory particles have also been described, such as 11S (PA 28) and PA 200, with different functions and activations as compared to the 19S RP. The 11S RP is involved in the immune-proteasome and is regulated by γ-interferon, whereas PA 200 RP is only present in the nucleus, although little is known about its specific function [26].

## 3. Mitochondria

**Figure 1:**
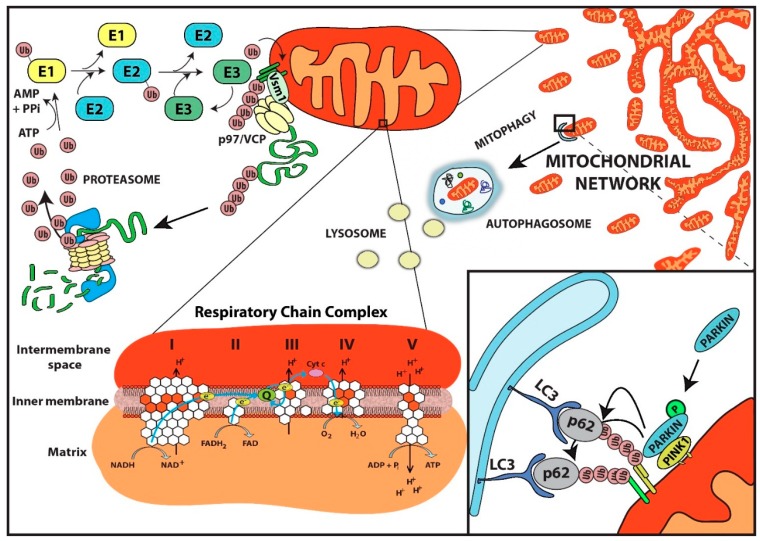
UPS and mitochondrial quality control. Polyubiquitination of mitochondrial proteins by the catalyzed reaction of E1, E2 and E3 enzymes in this depiction leads to the recruitment of the p97/VCP complex to the mitochondrial outer membrane (**upper left**). p97/VCP can extract a ubiquitinated protein in an ATP-dependent process that facilitates its proteasomal degradation. The UPS is also needed for the autophagic degradation of damaged mitochondria, a process known as mitophagy. Loss of mitochondrial membrane polarization stabilizes PINK1, which relocalizes to the outer membrane where it recruits and activates the E3 ligase PARKIN by phosphorylation. Once activated, PARKIN ubiquitinates several mitochondrial proteins, which flag the mitochondria for autophagic degradation (**lower right**). A schematic representation of the mitochondrial respiratory chain (complexes I, II, III, IV and V) is shown, with nuclear-encoded subunits depicted as white hexagons and the mitochondrial-encoded subunits as orange (**lower left**).

The endosymbiotic origin of mitochondria explains some of the unique biological aspects of these organelles [34], which form a dynamic network, often referred to as the mitochondrial network [35]. Mitochondria are regulated by fusion and fission, processes that are crucial to maintain functional mitochondria and energetic homeostasis. These processes, for example, enable small mitochondria to move along the cytoskeleton and relocate to areas where energy delivery is needed, such as the presynaptic terminals of an axon. In mammals, several proteins have been implicated in the regulation of fusion and fission of mitochondria. Mitofusin-1 and -2 (MFN1, MFN2) together with the optic atrophy 1 protein (OPA1) are required for mitochondrial fusion, while dynamin-related protein 1 (DRP1) is indispensable for fission [36,37]. All mitochondria contain two lipid bi-layers, an outer membrane (OMM) and an inner membrane (IMM), leading to the intermembrane space (IMS), chemically equivalent to the cytoplasm, and the matrix, an internal space that contains enzymes important for fatty acid oxidation as well as for the tricarboxylic acid (TCA), or Krebs cycle, as well as mtDNA. The IMM is highly impermeable, and by folding in a convoluted manner, forms the *cristae*, a large surface area where the respiratory chain (RC) complexes I–V are located (Figure 1).

Mitochondria are the only organelles that contain their own DNA. In humans, mitochondrial DNA (mtDNA) is a circular molecule that encodes 13 proteins, all of which are involved in OXPHOS, 22 transfer RNA species (tRNAs), and two ribosomal RNA types (16S, 12S). Each cell can contain several hundred copies of mtDNA (10^3^–10^4^ copies per cell) depending on the energy demand of the tissue, the differentiation stage of the cell, hormonal balance, and exercise level [38,39]. The vast majority of the ≈1000 mitochondrial proteins are encoded by nuclear genes [40], synthetized in the cytoplasm, and imported into the mitochondria in an unfolded state. During this process, cellular and mitochondrial chaperones (mtHSP70, mtHSP60, mtHSP10, *etc.*) assist the folding of imported proteins to ensure that they reach their destination to execute their function [41,42]. Mitochondria are the main source of reactive oxygen species (ROS), a natural by-product of OXPHOS. If not properly regulated, ROS can be extremely harmful to DNA, lipids and proteins, especially matrix proteins, which are not accessible by the cellular quality control machinery. In this regard, mitochondria possess their own quality control system consisting of several proteases, such as Lon, ClpXP, *i*-AAA, and *m*-AAA, to ensure that damaged or unfolded proteins that cannot be rescued and refolded by the mitochondrial chaperons are turned-over, thereby avoiding toxicity. Several reviews have been published on this topic [43,44,45]. The UPS is also an integral component of the mitochondrial protein quality control system, and mediates degradation not only of outer membrane embedded proteins, but also matrix proteins, implicating the existence of retro-translocation mechanisms of proteins from the mitochondrial matrix to the cytoplasm for proteasomal degradation [46].

## 4. Role of the Ubiquitin Proteasome System in Mitochondrial Protein Quality Control

The involvement of the UPS in the quality control of mitochondrial proteins started to emerge after several studies found components of the UPS in the mitochondria as well as ubiquitination of numerous mitochondrial proteins. In an early study conducted in yeast, the SCF ubiquitin ligase complex subunit Mdm30 (mitochondrial distribution and morphology protein 30) was shown to affect mitochondrial shape by regulating the steady-state level of Fzo1, an ortholog of mammalian mitofusin-1 and -2; thus connecting the ubiquitin proteasome system with mitochondria [47]. While attempting to determine the mitochondrial proteome of *Saccharomyces cerevisiae*, numerous E3 ligases and DUBs were found to be associated with the mitochondrial compartment [48]. In another study, the purification of total ubiquitinated proteins from mouse heart expressing 8xHis/Flag-Ubiquitin (HisF-Ub) under the α-myosin heavy chain (α-MHC) promoter, led to the finding that 38% of all ubiquitinated proteins were mitochondrial and found in all compartments, including the matrix [49]. One possible explanation for such findings could be that nuclear encoded mitochondrial proteins that are not properly folded during translation are directly targeted for degradation. In this regard, it has been estimated that one third of all synthetized proteins are defective ribosomal products (DRiPs), due to errors in transcription and/or translation, and are turned-over by the proteasome before reaching their final destination [50]. However, an interesting alternative possibility has been proposed: the existence of a mechanism to retro-translocate mitochondrial proteins into the cytosol for degradation, akin to the endoplasmic reticulum-associated degradation (ERAD) pathway, and thus named the mitochondria-associated degradation (MAD) system, also referred to as the outer mitochondrial membrane-associated degradation (OMMAD) system [51,52,53].

In support of the MAD process, it has been shown that colon cancer cells (COLO 205) treated with inhibitors of the chaperone protein, heat shock protein 90 (HSP90), undergo apoptotic cell death preceded by dramatic changes in the mitochondrial compartment [54]. The most prominent change was an accumulation of mitochondrial proteins due to an increase in protein half-life, as determined by ^35^S-methionine/cysteine pulse-chase. The authors found that one protein in particular, oligomycin-sensitivity-conferring protein (OSCP), which is a component of the mitochondrial membrane ATP synthase (F1F0-ATP synthase or complex V) and located in the IMM, was ubiquitinated and degraded by the proteasome in an HSP90-dependent manner [54]. Additionally, a role for ubiquitination and proteasome degradation has been described for the mitochondrial uncoupling protein 1 and 2 (UCP1, 2) as well as for the endonuclease G (endoG) protein [55,56,57]. Similarly with what has been described in the ERAD pathway, the Cdc48/p97 complex (cdc48: cell division control protein 48) seems to be required for the extraction of mitochondrial proteins in the MAD system [58]. In fact, it has been shown in yeast treated with mitochondrial stressors that the cytoplasmic protein Vms1 (valosin-containing protein (VCP)/Cdc48-associated mitochondrial stress-responsive 1) re-localizes to mitochondria and recruits the Cdc48/p97–Npl4 (Npl4: nuclear protein localization protein 4) complex (Figure 1) [52]. Interestingly, Vms1 overexpression in yeast has been shown to counteract the mitochondrial damage and cell death induced by the expression of UBB+1, a frame-shift variant of ubiquitin B, which is associated with Alzheimer’s disease [59]. Complex p97, known as VCP in mammals and Cdc48 in yeast, belongs to the ATPases associated with diverse cellular activities (AAA+) protein family, and is a barrel-shaped hexameric complex that uses ATP to unfold and extract proteins from membranes and protein complexes [23]. Notably, Cdc48/VCP mutations have been shown to induce a decrease in mitochondrial membrane potential and to increase mitochondrial oxygen consumption leading to mitochondrial damage and cell death both in yeast and human-derived fibroblasts [60,61].

Among the numerous E3 ligases associated with mitochondria, PARKIN is by far the most studied. Mutations in the PARK2 locus, where the *PARKIN* gene is located, were initially associated with autosomal recessive juvenile Parkinson’s disease (AR-JP) [62]. Further studies have contributed to understanding the function of PARKIN and the possible mechanism by which it might promote disease [63]. PARKIN has been described as an hybrid E3 ubiquitin ligase that possesses both RING and HECT E3 ligase characteristics [64]. Upon mitochondrial depolarization, the self-inactivated enzyme is thought to be recruited to the mitochondrial membrane where it is phosphorylated and activated by PTEN-induced putative kinase 1 (PINK1) [65]. PINK1 is constantly imported and degraded in healthy mitochondria; however, when perturbations of mitochondrial homeostasis affect the mitochondrial membrane potential, PINK1 escapes degradation and accumulates on the outer membrane. There, it recruits and activates PARKIN by phosphorylating the Ser65 residue of the PARKIN ubiquitin-like domain; however, its full activation also requires the phosphorylation of Ser65 on the ubiquitin molecule [66,67,68]. Once activated, PARKIN induces the removal of depolarized mitochondria by mitophagy through a poorly understood mechanism, which requires the poly-ubiquitination of several other outer membrane mitochondrial proteins, including MFN1 and 2, Mitochondrial Rho GTPase (RHOT)-1 and 2, and voltage-dependent anion channel (VDAC)-1, 2, and 3 [69,70,71,72]. Notably, up-regulation of Parkin in *Drosophila* resulted in increased mean and maximal lifespan, and was associated with reduced protein aggregation and improved mitochondrial activity in aged flies [73]. Although the PINK1/PARKIN pathway has been shown to be involved in the removal of depolarized mitochondria induced by stressors, such as carbonyl cyanide 3-chlorophenylhydrazone (CCCP), an uncoupler of oxidative phosphorylation, its involvement in the physiological removal of mitochondria seems to be nonessential, as demonstrated by the absence of striking phenotypes in Parkin and Pink1 knockout mice, thus suggesting the presence of additional mechanisms for the removal of mitochondria, independent of the PINK1/PARKIN pathway (reviewed in [74]). In fact, a study from our group showed that dysfunctional mitochondria in a mouse model for Parkinson’s disease generated by knocking out the mitochondrial transcription factor A (TFAM) in dopaminergic neurons, did not recruit PARKIN. Neither removal of defective mitochondria nor the neurodegenerative phenotype was affected by the absence of PARKIN in these mice [75].

Another RING/E3 ubiquitin ligase that seems to regulate mitochondrial dynamics is MITOL/MARCH-V (mitochondrial ubiquitin ligase), a membrane protein located in the OMM where it interacts with and ubiquitinates several substrates [76]. One such substrate is Drp1, which is degraded upon MITOL-mediated ubiquitination; thus, MITOL might affect mitochondrial fission by regulating Drp1 levels [77,78]. Furthermore, MITOL seems to be involved in the ubiquitination and degradation of misfolded proteins located in mitochondria, such as a mutated form of superoxide dismutase 1 (SOD1), an antioxidant enzyme that has been implicated in amyotrophic lateral sclerosis (ALS, or Lou Gehrig’s disease) [79]. Additionally, several DUBs have been localized to mitochondria, such as ataxin-3, a deubiquitinating enzyme that is associated with Machado-Joseph disease and seems to interact with PARKIN in order to counteract self-ubiquitination [80].

Taken together, these studies support a central role for the UPS in the maintenance of mitochondrial homeostasis by regulating organelle dynamics (fission and fusion), the proteome, and mitophagy. Thus, it is not surprising that disturbances affecting UPS activity might also have an effect on mitochondrial function. With that said, studies also support that the converse is also true.

## 5. Effect of Mitochondrial Dysfunction on the Ubiquitin Proteasome System

Evidence that mitochondrial dysfunction might affect proteasomal activity has been reported in different systems, including yeast, *C. elegans*, and mammalian cells. It has been shown that inhibition of OXPHOS in rat-derived cortical neurons also affects proteasomal activity and protein ubiquitination [81]. Two recent reports have helped to shed light on the possible molecular mechanisms underlying such an effect [82,83]. Stimulation of ROS production in a respiration-deficient yeast mutant (*Δfzo1*) was shown to induce proteasome disassembly, with the complete detachment of the 20S CP and 19S RPs, similar to what was observed in yeast and mammalian cells treated with either hydrogen peroxide (H_2_O_2_) or antimycin A, a cytochrome *c* reductase inhibitor. Proteasome disassembly was associated with proteasomal substrate accumulation and was reversed upon treatment with antioxidants or dithiothreitol (DTT), a strong reducing agent [82]. Comparable results were obtained in a different study, using a short-lived ubiquitin fused protein expressed in *C. elegans* as a reporter, to screen for factors involved in regulating protein turnover. Screening revealed reporter accumulation in two worm mutants carrying mutations in proteins involved in mitochondrial processes: IVD-1 and ACS-19. IVD-1 is the ortholog of a human mitochondrial enzyme (isovaleryl-CoA dehydrogenase) involved in the leucin catabolism pathway, while ACS-19 is predicted to be the ortholog of a human enzyme (ACSS2, acetyl-CoA synthetase) involved in fatty acid metabolism in the mitochondrial matrix. In both cases, the effect of mitochondrial dysfunction on proteasomal function was due to an increase in ROS production, which was prevented by treatment with the antioxidant *N*-acetylcysteine (NAC) [83].

**Figure 2:**
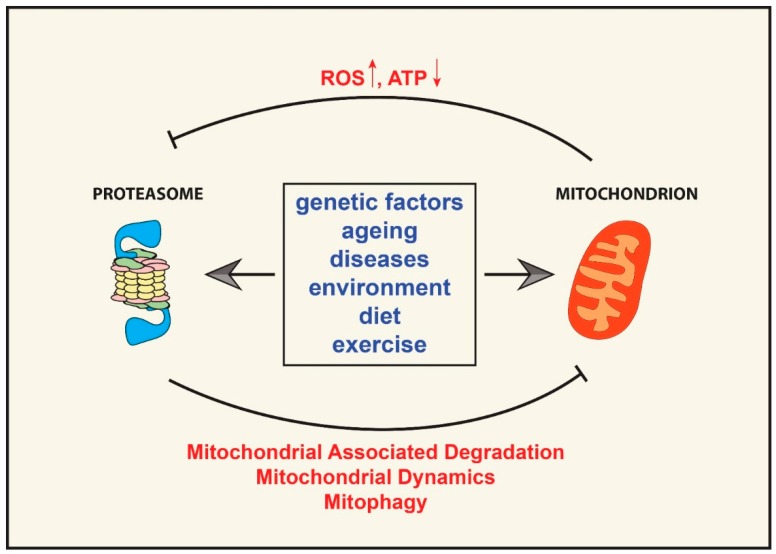
UPS and mitochondrial cross-talk. Several factors, including genes, environment, age, diseases, diet, and exercise can either positively or negatively affect UPS activity and mitochondrial function. Impairment of one of the two systems can then drive the malfunctioning of the other and result in a vicious cycle. A decrease in cellular ATP levels and an increase in ROS production can impair proteasomal function by affecting protein ubiquitination and proteasome assembly and stability, while a decrease in UPS activity could impair mitochondrial function by affecting mitochondrial dynamics, mitophagy, and the removal of damaged mitochondrial proteins.

ROS is a group of potentially harmful compounds that can damage all cellular components, including proteins, DNA, and lipids. Oxidation can affect protein structure, thus impairing function, and might also render proteins prone to aggregation, which could result in toxicity. The complete disassembly of the proteasome, resulting in an increase of 20S CPs, could be a protective mechanism to counteract a temporary rise in oxidative damage. It has been shown that 20S CP is more resistant to oxidative damage, compared to 19S RP, and is able to bind and degrade mis-folded oxidized proteins without the need for ubiquitination and ATP expenditure [84,85,86]. Thus, a temporary disassembly of the proteasome holoenzyme together with an up-regulation of an antioxidant stress response, heat shock proteins, and autophagic flux could be seen as part of a cellular strategy to counteract an acute increase in oxidative damage. Hence, through the uncapping of the 20S CP, cells might redirect the degradation capability of the proteasome from the removal of ubiquitinated substrates to the removal of oxidized proteins. However, since oxidative stress is a hallmark of ageing and age-related diseases, chronic exposure to oxidative stress could result in proteasome disassembly, which could further aggravate these conditions (Figure 2).

ATP depletion is another mechanism through which mitochondrial dysfunction might affect proteasomal activity. ATP is required for both protein ubiquitination [87] and proteasome assembly and stability [88,89,90]. Intracellular ATP levels have been shown to regulate proteasomal activity both *in vitro* and in cultured cells [91], and manipulation of intracellular ATP levels by inhibition of complex I has been shown to decrease proteasomal activity in primary mesencephalic cell cultures, an effect which was counteracted by increasing the glucose concentration in the cellular medium [92].

## 6. The “Mitochondrion—Ubiquitin Proteasome System Axis” in Ageing and Age-Related Diseases

The UPS and mitochondria are two systems among several reportedly affected by ageing; an accumulation of mis-folded proteins and oxidative stress have been denoted as two features of the ageing process. A decline in UPS activity has been shown in yeast (*Saccharomyces cerevisiae*) [93], fly (*Drosophila melanogaster*) [94], rodents [95,96,97], and also in human-derived dermal fibroblasts [98]. Conversely, it has been shown that proteasome activation by genetic manipulation in different models can ameliorate the ageing process and also increase lifespan (reviewed in [99]). Several possibilities have been proposed to explain the UPS decline associated with ageing, including down-regulation and/or modification of proteasomal subunits, disassembly of the holoenzyme, an increase in substrates and aggregates that could clog the proteasome, and reduction in ATP levels, which could impair the overall process of protein ubiquitination and unfolding [100]. As mentioned, an increase in oxidative damage is a major contributor to the UPS decline, and with OXPHOS as the main source of ROS production, mitochondria have thus been suspected to play a central role in the ageing process. Based on this notion, Denham Harman proposed the “Free Radical Theory of Aging” (FRTA) in 1956, suggesting that ageing is driven by the accumulation of oxidative damage to cellular structures over time [101]. It has been proposed that accumulation of mtDNA mutations could be a possible cause of the mitochondrial dysfunction described in ageing, and in this regard data from different groups, including ours, have shown a cause-effect relationship between increased mtDNA mutational load and ageing phenotypes [102,103,104,105,106,107,108,109,110]. However, it has also been argued that the level of mtDNA mutations observed in normally aged tissues is much less than the threshold needed to cause respiratory chain dysfunction [111,112]. Thus, another possibility for the age-associated decline in mitochondrial function could be a loss in protein homeostasis due to the impairment of the UPS and/or autophagic systems.

As described, the UPS and mitochondria systems are tightly interdependent, and once a vicious cycle of dysfunction starts it is difficult to identify which one was the trigger (Figure 2). This is demonstrated in neurodegenerative diseases, such as Alzheimer’s disease (AD) and Parkinson’s disease (PD), with ageing consistently implicated as the major risk factor. In both diseases, it has been seemingly difficult to isolate UPS impairment from dysfunctional mitochondria, and *vice versa*, in order to understand the contribution of each system in disease onset and progression. PD is a neurodegenerative disorder that arises from the loss of dopaminergic neurons, mainly in substantia nigra, and is characterized by resting tremor, bradykinesia, and muscle rigidity. The discovery of Lewy bodies in neurons, aggregates containing α-synuclein, ubiquitinated proteins, and components of the UPS, strongly implicated the proteasome in the pathogenesis of the disease [113]. However, other studies have reported a compelling correlation between mitochondrial dysfunction and PD, and mouse models mimicking the disease have been generated by genetically impairing mitochondrial function in dopaminergic neurons [114] or by using toxins that affect mitochondria, such as 1-methyl-4-phenyl-1,2,3,6-tetrahydropyridine (MPTP) [115]. In all likelihood, PD will turn out to be several different diseases characterized by different etiologies, although only partially different phenotypes. AD patients exhibit gross brain atrophy, with both neuronal and synaptic loss, accumulation of amyloid plaques containing amyloid β peptides, and intracellular neurofibrillary tangles of phosphorylated Tau protein [116]. The involvement of the UPS in AD has been postulated based on studies demonstrating a decrease in proteasome activity associated with AD and the presence of ubiquitin and UPS components in the plaques [117]. As similarly shown with PD, another body of literature has focused on mitochondrial dysfunction as representing the major etiopathogenesis of AD [118]. Taking both perspectives into consideration, perhaps these two interconnected systems should be regarded as the “Mitochondrion-UPS Axis” when trying to understand and dissect the cellular dysfunction observed in ageing and age-related diseases. That is, UPS impairment and mitochondrial dysfunction could be two sides of the same coin in that either system cannot be separated from the other since they affect each other in a vicious cycle (Figure 2).

**Figure 3:**
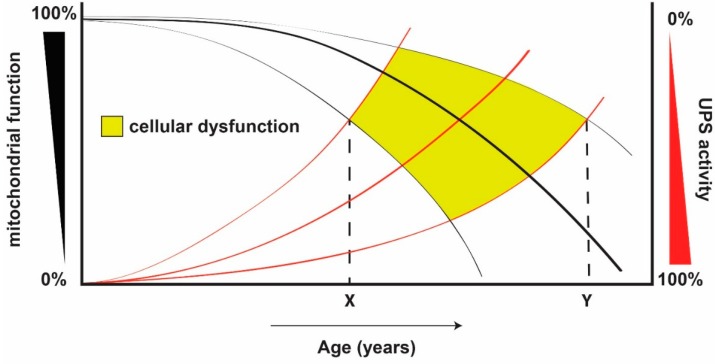
Hypothetical model to explain the heterogeneity of the ageing process among individuals taking into consideration changes in both UPS and mitochondrial function. A theoretical model to explain the idiosyncratic differences observed during ageing by taking into consideration the variation of both UPS activity and mitochondrial function over time. Both systems decline with age in a dependent manner, but the slope of the curve may vary between individuals, depending on factors such as genetics, environment, diet, and exercise, as depicted by the thin lines. The point of interception of the two curves hypothetically represents the age of onset of cellular dysfunction, defined as any point in time when cellular homeostasis is perturbed. Thus, two individuals, each following different extremities of mitochondrial dysfunction and UPS decline, might experience the onset of cellular dysfunction at different ages (X and Y), which could be decades apart from each other.

In order to explain the differences observed among individuals during ageing, we propose a model that takes into consideration the decline in both mitochondrial function and UPS activity over time. We speculate that the point of interception between the two systems might represent the age at which cellular dysfunction begins (Figure 3). While both systems decline with age in a dependent manner, the shape of each curve will vary slightly between individuals, due to the compounded effects of an individual’s genetic background, environmental stressors (*i.e*., toxins, smoking), diet, and exercise. Taking these factors into account, the age of cellular dysfunction onset for a given person could start decades earlier as compared to another, leading to the ageing heterogeneity of the human population.

## 7. Conclusions and Future Prospects

The last century has witnessed a considerable increase of life expectancy due to better living conditions and medical advancements in the cure and prevention of many once fatal diseases. Several compounds, such as resveratrol, metformin, and rapamycin, have shown potential in improving overall health and lifespan in experimental organisms. Finding drugs to combat ageing might therefore not be just fantasy, but actually feasible [119,120]. However, the only currently known proven interventions shown to improve ageing phenotypes in humans are a hypocaloric diet and exercise [121,122]. Therefore, understanding the underlying molecular mechanism of the ageing process is the *condicio sine qua non* for developing any promising therapeutic intervention to slow the ageing process. In this regard, we suggest that dissecting the “Mitochondrion-UPS Axis” may help in the search for drugs to counteract ageing and age-related diseases.

## Abbreviations

AD = Alzheimer’s Disease; DUBs = Deubiquitinating enzymes; HECT = Homologous to the E6-AP Carboxyl Terminus; IMM = Inner Mitochondrial Membrane; IMS = Intermembrane Space; RC = Respiratory Chain; MAD = Mithochondrial Associated Degradation; OMM = Outer Mitochondrial Membrane; OXPHOS = Oxidative Phosphorylation; PD = Parkinson’s Disease; RING = Really Interesting New Gene; ROS = Reactive Oxygen Species; Ub = Ubiquitin; UPS = Ubiquitin Proteasome System.
